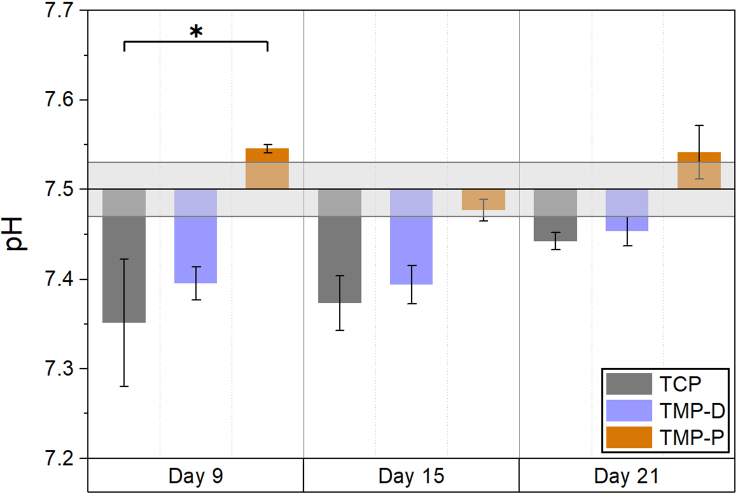# Degradation of 3D-printed magnesium phosphate ceramics *in vitro* and a prognosis on their bone regeneration potential

**DOI:** 10.1016/j.bioactmat.2022.04.015

**Published:** 2022-04-26

**Authors:** Gefel Eugen, Moseke Claus, Schmitt Anna-Maria, Dümmler Niklas, Stahlhut Philipp, Ewald Andrea, Meyer-Lindenberg Andrea, Vorndran Elke

**Affiliations:** a Institute and Department for Functional Materials in Medicine and Dentistry, University Clinic Wuerzburg, Wuerzburg, Germany; b Institute for Biomedical Engineering (IBMT), University of Applied Sciences Mittelhessen (THM), Wiesenstraße 14, Gießen, Germany; c Clinic for Small Animal Surgery and Reproduction, Ludwig-Maximilians-Universität, Munich, Germany

**Keywords:** Newberyite, Struvite, Bioresorption, Ceramic bone implants, Human osteoclasts, Bioactivity

## Abstract

Regenerative bone implants promote new bone formation and ideally degrade simultaneously to osteogenesis. Although clinically established calcium phosphate bone grafts provide excellent osseointegration and osteoconductive efficacy, they are limited in terms of bioresorption. Magnesium phosphate (MP) based ceramics are a promising alternative, because they are biocompatible, mechanically extremely stable, and degrade much faster than calcium phosphates under physiological conditions. Bioresorption of an implant material can include both chemical dissolution as well as cellular resorption. We investigated the bioresorption of 3D powder printed struvite and newberyite based MP ceramics *in vitro* by a direct human osteoclast culture approach. The osteoclast response and cellular resorption was evaluated by means of fluorescence and TRAP staining, determination of osteoclast activities (CA II and TRAP), SEM imaging as well as by quantification of the ion release during cell culture. Furthermore, the bioactivity of the materials was investigated via SBF immersion, whereas hydroxyapatite precipitates were analyzed by SEM and EDX measurements. This bioactive coating was resorbed by osteoclasts. In contrast, only chemical dissolution contributed to bioresorption of MP, while no cellular resorption of the materials was observed. Based on our results, we expect an increased bone regeneration effect of MP compared to calcium phosphate based bone grafts and complete chemical degradation within a maximum of 1.5–3.1 years.

## Highlights

• 3D powder printing allows individual shaping of porous magnesium phosphate bone implants.
• Precipitation of amorphous hydroxyapatite confirmed the bioactivity of magnesium phosphate ceramics.
• The compatibility of magnesium phosphates with human osteoblasts and osteoclasts could be verified in a direct culture approach. Whereat, TRAP and CA II activity were in the same range or slightly increased compared to osteoclasts on β-tricalcium phosphate.
• 3D powder printed magnesium phosphates showed no sign of osteoclastic resorption*,* but a continuous chemical degradation.

## Graphical abstract

**Figure:**
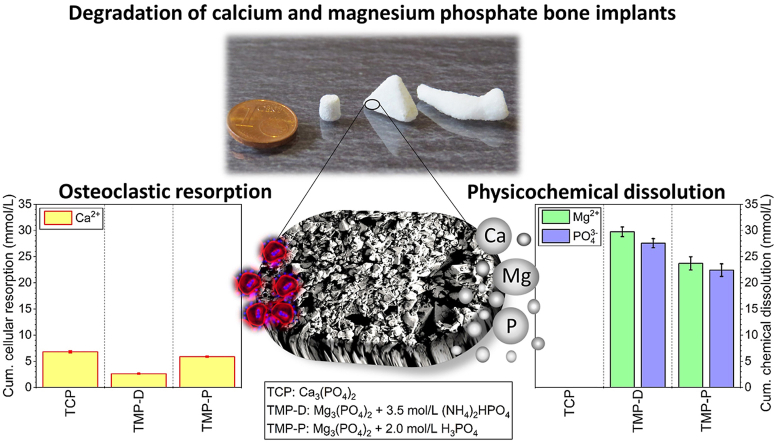

## 1 Introduction

Bone is one of the few tissues which have the inherent ability to regenerate in the sense of a *restitutio ad integrum*. However, the regenerative capacity of bone tissue is limited to a certain defect size, above which the body is no longer able to fuse the defect ends by a successive ingrowth of bone tissue. This so-called critical size is already reached for bone defects above 5 cm^3^ [1]. Besides this, several patient-related as well as independent risk factors can negatively impair bone regeneration [1]. Under these circumstances, ingrowth of fibrous tissue or cartilage is a frequently occurring complication, which inevitably results in a non-union of bony defect edges and hence in biomechanical instability. To avoid this, alloplastic materials have become clinically established for the treatment of critical size bone defects, in addition to the use of native bone tissue of autologous, allogeneic or xenogeneic origin [2,3]. Numerous advantages, including unlimited availability, sterilizability, and the lack of donor side morbidity, pathogenic transfer or immunogenic complications, make the use of synthetic bone graft materials attractive for clinicians and still arouse great interest in research regarding their potential for optimization [1], even more since most synthetic materials can be processed using additive manufacturing methods, which provide the possibility of creating anatomically customized implants [4,5]. The currently available synthetic bone substitutes on the market comprise bioinert non-resorbable metals like titanium, non-resorbable ceramics like alumina or zirconia, and polymers like polymethyl methacrylate, as well as bioceramics, e.g. calcium phosphate ceramics (CPC) and bioglass or silica-based ceramics [6,7]. The latter ones are characterized by the ability to form low crystalline carbonated hydroxyapatite (CHA) on their surface in contact with native biological fluids or serum-like solutions, which is generally defined as *bioactivity* [8,9]. The precipitation of this bone-like apatite layer is associated with the bone bonding ability of these materials, resulting in a mechanically stable osseointegration [10]. Moreover, their mineral composition and porous structure promote *de novo* bone formation or even induce bone regeneration [[11], [12], [13]]. These attractive properties have made bioceramics established materials in clinical applications. In particular, hydroxyapatite (HA) and tricalcium phosphate (TCP) are the most commonly applied bioceramic bone substitutes [14,15]. In addition to pastes, granules, or monoliths, bioceramics are used to coat bioinert implants to prevent fibrous encapsulation and to improve their bone bonding ability [7,8]. Nevertheless, compared to autografts, currently implanted bioactive materials, e.g. HA [15,16] or bioglass [7] show a limitation in terms of their regenerative effect [15,16], due to their long term *in vivo* stability. The implant residues are associated with an increased risk of inflammation or - due to the brittle nature of ceramics - fractures of newly formed bone [2]. To overcome these limitations, novel synthetic bone substitutes are being developed with respect to their potential for their complete replacement by natural bone tissue. A fundamental requirement for this is the degradation of the synthetic material with degradation kinetics corresponding to the rate of osteogenesis [17]. Bioresorption can include both chemical dissolution as well as cellular resorption of the material [18], and it is generally considered to be an important requirement for an ideal bone implant [19]. While solubility is a material-inherent chemical property and depends on environmental conditions, like pH, ion composition, and temperature, cellular resorption can be performed by macrophages and osteoclasts [20,21]. Macrophages, as part of the host immune response, usually remove foreign material by phagocytosis, which includes particles <20 μm [22] of synthetic materials [20]. These cells are particularly active in the early phase (day 1–7) of the bone healing cascade and are mainly responsible for the degradation of quick-dissolving materials [23]. Osteoclasts, on the other hand, are by nature specialized in the resorption of materials that correspond to components of native bone, in particular CHA and proteins. This enables osteoclasts to produce bone cavities with a migration rate of 50–100 μm/day [23]. To dissolve inorganic and organic components they secrete hydrochloride acid and proteolytic enzymes such as cathepsin K [24]. Osteoclast-mediated resorption is a very slow process and occurs at a late stage of the bone healing cascade [25]. Besides bone resorption osteoclasts play a pivotal role in bone remodelling, due to the transcellular release of calcium ions and their interaction with osteoblasts in the basic multicellular unit [24,26]. Consequently, even in the case that osteoclasts are not involved in the resorption of an implant material, their adhesion, activation or inhibition affects the process of bone regeneration. Known materials that are not resorbed by osteoclasts, are dicalcium phosphate dihydrate [20], bioglass, and various synthetic polymers [27]. Since chemical and structural properties of the implant material (e.g. composition, solubility, crystallinity, and porosity) affect osteoclast adhesion and resorption activity [21], the knowledge of the compatibility between osteoclasts and a bone graft material is essential for the estimation of its bone healing efficacy.

During the last years, the interest in completely bioresorbable magnesium-based bone implants has been increasing significantly. This involves magnesium alloys [28] as well as magnesium phosphate (MP) ceramics [19,29]. The elastic moduli of magnesium and its alloys are close to that of cortical bone; furthermore magnesium is considered to be biocompatible, osteoinductive, and angiogenic [28]. However, the *in vivo* degradation of the metal still poses a challenge, since it is usually occurring too quickly and is based on corrosion, which involves the formation of hydrogen gas at the peri-implant sites [28]. For this reason, MP is considered to be superior in terms of degradation, as it proceeds *in vivo* almost simultaneously with bone regeneration and is not associated with hydrogen gas release [30]. As compared to CPC, MP show a higher solubility [31], with the release of Mg^2+^ ions stabilizing the formation of amorphous CHA under physiological conditions and inhibiting the transformation into poorly soluble HA crystals [32]. Studies on MP are still limited and most often focused on sintered trimagnesium phosphate (farringtonite, Mg_3_(PO_4_)_2_) or struvite (NH_4_MgPO_4_·6H_2_O)-forming cements [19,33]. The latter one uses farringtonite or magnesium oxide as educts, which react with an alkaline solution (e.g. di-ammonium hydrogen phosphate or potassium hydrogen phosphate solution) to form struvite [33] or k-struvite (MgKPO_4_·6H_2_O) ceramics [34]. Only a few studies deal with newberyite, a hydrated MP, formed by the reaction of farringtonite or magnesium oxide with acidic phosphate solutions [31]. Despite the limited *in vivo* and *in vitro* studies, the biocompatible, osteoconductive, and osteointegrative properties of MPs have been confirmed, as no inflammatory rejection or foreign body reactions were observed and healing occurs without the formation of connective tissue [[29], [30], [31]]. In the few studies that have been conducted it was shown that degradation of MP took place over a period of 3–6 months and the successive replacement of the ceramics by bone resulted in a complete conversion of the implant to native bone tissue [20,25]. To what extent osteoclasts are involved in this process is still not well analyzed and has not yet been elucidated at all for newberyite.

For this purpose, we investigated the bioactivity and bioresorption of 3D-printed acidic or alkaline treated MP *in vitro* in order to evaluate the longevity of struvite- and newberyite-based bone implants and their osteogenic potential in comparison to the clinically established and commonly used TCP*.* In particular, we focused on the questions to what extent human osteoclasts are involved in the degradation process of these ceramics by using a direct cell cultivation approach and in which way the specific degradation of MP will influence bone regeneration. In this context, also the effects of the MP-based ceramics on the differentiation of monocytes into osteoclasts was analyzed.

## 2 Materials and methods

### 2.1 Ceramic cement powder preparation

Ceramic powder batches of either Ca_3_(PO_4_)_2_ or Mg_3_(PO_4_)_2_ (TMP) were synthesized by sintering blends of 2 mol calcium hydrogen phosphate (CaHPO_4_, J.T. Baker, Philipsburg, USA) and 1 mol calcium carbonate (CaCO_3_, Merck, Darmstadt, Germany) or 2 mol magnesium hydrogen phosphate (MgHPO_4_·3H_2_O, Fluka, Altmann Analytik GmbH & Co. KG, Munich, Germany) and 1 mol magnesium hydroxide (Mg(OH)_2_, VWR International GmbH, Darmstadt, Germany), respectively. While Mg_3_(PO_4_)_2_ was sintered at 1100 °C, Ca_3_(PO_4_)_2_ was sintered at 1400 °C. In each sintering process the temperature was maintained for 5 h. After cooling the sintered cakes were manually crushed with mortar and pestle, followed by grinding in portions of 125 g in a 500 mL zirconia jar with 4 zirconia balls (30 mm) in a planetary ball mill (PM400 Retsch, Haan, Germany) at 200 rpm for 10 min. The cement synthesis was finished by sieving the powders to a particle size <355 μm.

### 2.2 Scaffold fabrication and characterization

All ceramic scaffolds were fabricated in a multi-step process. This process included 3D powder printing of a polymer-ceramic blend and sintering of printed specimens. In addition, TMP scaffolds were infiltrated with reactive solutions, followed by a rinsing step. The cement solidification during 3D printing was based on polymer swelling. Therefore, Ca_3_(PO_4_)_2_ or Mg_3_(PO_4_)_2_ ceramic powders were blended with 4 wt% hydroxypropyl methylcellulose (HPMC, Sigma-Aldrich, Steinheim, Germany) before printing. Disc-shaped ceramic scaffolds (h = 2 mm, Ø = 5.5 mm) were printed with the HPMC blended ceramic powders and water as printing solution in a 3D powder printer Z310 (Z-Corporation, Burlington, USA). A layer thickness of 100 μm and a binder/volume ratio of 0.275, equal for the core and the shell of the scaffold, were set as printing parameters. After printing, the scaffolds were removed from the powder bed and gently rid of powder residues using compressed air. Thereafter the scaffolds underwent a heat treatment according to an appropriate programme (Table 1), which included a debinding step (sintering plateau 1) and an individual final sintering temperature (sintering plateau 2) depending on the cement type. After the sinter process the TMP samples were either immersed for 24 h in a 3.5 M (NH_4_)_2_HPO_4_ solution (1 mL/scaffold, hereinafter referred to as TMP-D) or soaked twice with 1 M H_3_PO_4_ (hereinafter referred to as TMP-P). Powder printed TCP scaffolds were used as ceramic reference without any additional treatment with reactive solutions.

**Table 1:** Sintering programmes for ceramic scaffolds.

Cement type	Heating rate 1	Sintering plateau 1	Heating rate 2	Sintering plateau 2
Mg_3_(PO_4_)_2_	120 °C/h	500 °C, 2 h	300 °C/h	1200 °C, 4 h
Ca_3_(PO_4_)_2_	120 °C/h	500 °C, 2 h	300 °C/h	1350 °C, 4 h

Previous to gamma sterilization (31 kGy) the cell culture scaffolds were washed twice in sterile distilled water (dH_2_O) and twice in phosphate-buffered saline (PBS, 8.00 g NaCl, 0.20 g KCl, 0.27 g KHPO_4_, 1.43 g NaHPO_4_ in 1 L dH_2_O). For this purpose, the scaffolds were placed completely covered by the washing solution in a petri dish on a tilt shaker. The washing medium was exchanged every 24 h. For each analysis powder printed scaffolds composed of clinically established Ca_3_(PO_4_)_2_ were used as reference material.

The chemical composition of the raw cement powders and the sterile scaffolds was determined by X-ray diffraction (XRD) using a diffractometer D8 Advance (Bruker Corporations, Karlsruhe, Germany) with monochromatic Cu-Kα radiation. Data was collected from 2θ = 10–40° with a step size of 0.02°, a normalized count time of 0.5 s/step, and under rotation of the powder cuvette with 15 rpm. The phase composition was checked by means of JCPDS reference patterns for ß-Ca_3_(PO_4_)_2_ (β-tricalcium phosphate, β-TCP, PDF Ref. 09-0169), α-Ca_3_(PO_4_)_2_ (α-tricalcium phosphate, α-TCP, PDF Ref. 09-0348), Mg_3_(PO_4_)_2_ (farringtonite; PDF Ref. 33-0876), MgHPO_4_·3H_2_O (newberyite, PDF Ref. 35-0780), Ca_9_(PO_4_)_5_OH (hydroxyapatite, PDF Ref. 46-0905), and NH_4_MgPO_4_·6H_2_O (struvite, PDF Ref. 15-0762). On the basis of the recorded XRD patterns, a quantitative phase analysis was performed according to the Rietveld-Refinement method using the software TOPAS V6 (Bruker Corporation, Billerica, USA). For the quantification of open porosity and the determination of the pore size distribution a mercury porosimeter (Pascal 140/440, Thermo Fisher Scientific Inc., Waltham, USA) was used. Each scaffold type was examined 3 times (*n* = 3) with regard to the pore structure. For each measurement 0.1–0.3 g of printed scaffolds were used. The porosity was recorded in a pressure range from 0.01 kPa to 400 MPa and the data was evaluated by the software SOLID (SOLver of Intrusion Data Ver. 1.6.5, Thermo Fisher Scientific Inc. Waltham, USA). The surface topography of the ceramic scaffolds was described on the basis of the pore and particle size distributions. The size of particles and agglomerates was measured with the software ImageJ (Version 1.53 m, Rasband, W.S., ImageJ, U. S. National Institutes of Health, Bethesda, Maryland, USA) on the basis of field emission scanning electron microscope (FESEM) images in top surface view of washed sterile scaffolds. Prior to FESEM analysis the specimens were coated with platinum using a sputter coater (Leica EM ACE600, Leica Mikrosysteme GmbH, Wetzlar, Germany). The FESEM images were recorded with a CB 340 Zeiss FESEM (Zeiss, Oberkochen, Germany) in secondary electron mode.

### 2.3 Bioactivity testing with simulated body fluid

Cylindrical samples (Ø = 5 mm, h = 5 mm, n = 5) prepared as previously described, were immersed separately in 2 mL of a simulated body fluid (SBF)-JL2 solution [10] in wells of a cell culture well plate. The SBF solution was prepared according to Bohner et al. [10]. In brief, a solution A (6.129 g/L NaCl, 5.890 g/L NaHCO_3_, 0.498 g/L Na_2_HPO_4_·2H_2_O, and 0.934 mL/L 1.00 M HCl in H_2_O, pH = 7.4) and a solution B (6.129 g/L NaCl, 0.540 g/L CaCl_2_, 0.934 mL/L 1.00 M HCl in H_2_O, pH = 7.4) were stored in separate syringes, preconditioned at 37 °C, and mixed by connecting the syringes via a luer lock just before starting the bioactivity experiment. The bioactivity test was performed at 37 °C, under 5% CO_2_ atmosphere, and no stirring over a period of 7 days. After 7 days the specimens were removed from the solution, gently washed with distilled water, and dried at 37 °C. The surfaces of the specimens were examined for HA precipitates with FESEM after sputter coating with platinum as described previously (2.2). In addition to topographical examination, the distribution of magnesium, calcium, phosphorous, and carbonate was analyzed by means of energy dispersive X-ray spectroscopy (EDX) for MP ceramics, using an INCA Energy 350 AzTec Advanced system with silicon drift detector (Oxford Instruments, Abingdon, UK) and an acceleration voltage of 10 KV. Moreover, phase composition of the scaffolds after SBF immersion was determined by XRD analysis as described previously (2.2).

### 2.4 Cytotoxicity testing

The cytocompatibility of ceramic materials was evaluated in a direct cell culture approach with the human fetal osteoblastic cell line hFOB1.19 (ATCC® CRL11372™, Rockville, MD, USA). One day before cell seeding, each sterile ceramic scaffold was placed in a well of a 96-well plate and preconditioned in 200 μL of a 1:1 mixture of Gibco Dulbecco's Modified Eagle Medium (DMEM) and Ham's F12 Medium (Gibco®, Life Technologies, Darmstadt, Germany) modified with 100 U/mL Penicillin and 100 μg/mL Streptomycin (P/S, Gibco®, Life Technologies, Darmstadt, Germany), 20 mM HEPES (Sigma Aldrich, Taufkirchen, Germany), 10% fetal calf serum (FCS, Sigma Aldrich, Taufkirchen, Germany), and Geneticin G 418 Sulphate (Gibco®, Life Technologies, Darmstadt, Germany) in a concentration of 0.34 mg/mL at 34 °C in a 5% CO_2_ atmosphere. The medium was aspirated on the day of cell seeding and 1.2 × 10^4^ cells in supplemented DMEM/F12 medium were plated on each TCP, TMP-D, or TMP-P scaffold and cultured for 7 d at 34 °C and 5% CO_2_. The number of cells seeded per cm^2^ is shown in Table 2. TCP as established bone replacement was used as reference material to evaluate cell reactions based on material composition. Powder printing of TCP scaffolds guaranteed a comprehensibility with respect to the manufacturing method. Cells of the passage 15 were used for cell experiments. At day 1, 3, and 7 cell activity and cell number were quantified and live/dead staining was performed. The culture medium was exchanged at day 3 and day 5. The cell activity was quantified by means of WST assay using WST-1 reagent (Roche Diagnostics, Mannheim, Germany). Therefore, cell-seeded scaffolds were incubated with a 1:10 dilution of WST-1 reagent in modified DMEM/F12 at 34 °C and 5% CO_2_. After 30 min the metabolic cell activity was quantified by absorption measurements of the supernatants in a Tecan plate reader at 450 nm (Spark 20 M, Tecan, Crailsheim, Germany). Cell number on the scaffolds was quantified using the Quant-iT PicoGreen Kit (Thermo Fisher Scientific, Darmstadt, Germany) according to the manufacturers' protocol. In brief, cells on the scaffolds were washed twice in 0.2 M carbonate buffer, followed by cell lysis in 200 μL 0.1% Triton X-100 (Sigma Aldrich, Taufkirchen, Germany) in 0.2 M carbonate buffer on ice for 60 min. The cell lysis was completed after 3 freeze-thaw cylces of the lysate. 10 μL of the cell lysates and 190 μL of Quanti-iT PicoGreen reagent were incubated for 5 min in wells of a black 96-well plate under rotation (100 rpm) at RT in the dark. The fluorescent intensity was measured using the Tecan plate reader with an excitation wavelength of 485 nm and an emission wavelength of 535 nm. The DNA concentration was quantified according to a standard calibration curve. The DNA content was used to calculate the proliferation rate. The activity of the cells cultured on different surfaces was presented as WST-1 activity per pg DNA content. According to ISO 10993-5, the calculation of the cell viability was based on the optical density of cell culture supernatants recorded during WST assay [35].

**Table 2:** Definition of cell culture materials and seeded cell number per cm^2^. TCP as established bone replacement was used to evaluate material-depended cell reactions. Powder printing of TCP guaranteed a comprehensibility with respect to manufacturing method. For monocyte/osteoclast cell culture PS and glass was used as established cell culture control.

	Cell culture material				
	Experimental materials		Reference materials	Cell culture control	
	TMP-P	TMP-D	TCP	PS	glass
Cytotoxicity study osteoblast per cm^2^	4.0 × 10^4^	4.0 × 10^4^	4.0 × 10^4^	-	-
Cellular resorption study monocytes per cm^2^	6.6 × 10^5^	6.6 × 10^5^	6.6 × 10^5^	3.3 × 10^5^	3.3 × 10^5^

In addition, a viability staining of hFOB cells attached to the scaffolds was performed using fluorescent staining with the live/dead viability kit for mammalian cells (Thermo Fisher Scientific, Darmstadt, Germany). In brief, cells on the scaffolds were incubated in 200 μL hFOB culture medium modified with the fluorescent dyes calcein AM (2 μM) and ethidium homodimer-1 (1 μM) for 30 min in the dark. Imaging was performed with a fluorescence microscope Axio Observer (Carl Zeiss AG, Oberkochen, Germany) at an excitation wavelength of 494 nm or 528 nm and an emission wavelength of 517 nm or 617 nm for calcein AM (viable cells; stained green) or ethidium homodimer-1 (dead cells; stained red) staining. The percentage of living cells was determined by counting the number of living and dead cells in the fluorescence-stained cell culture. The software ImageJ was used for this purpose, whereas 2 images per sample *n* forming the basis of the calculation (*n* = 2).

### 2.5 Osteoclast cell culture and characterization

#### 2.5.1 Monocyte isolation, cell seeding and osteoclast differentiation

##### 2.5.1.1 Monocyte isolation

Human monocytes were isolated from a buffy coat (BRK, Wiesentheid, Germany) by density gradient centrifugation [36,37] involving 3 steps: 1. Isolation of peripheral blood mononuclear cells (PBMC) on a Pancoll density gradient, 2. Separation of monocytes and lymphocytes with a hyper-osmotic Percoll solution, and 3. Separation of monocytes from thrombocytes with an iso-osmotic Percoll solution. In detail, 22.5 mL human Pancoll solution (1.077 g/L, PAN-Biotech, Aidenbach, Germany) were filled into a 50 mL plastic tube and overlaid with a 1:2 dilution of the buffy coat (1:1 mixture of buffy coat with a modified PBS solution (E/B-PBS, PBS with 2 mM ethylenediaminetetraacetic acid (EDTA) and 0.5% Bovine Serum Albumin (BSA) (both Sigma Aldrich, Taufkirchen, Germany)) to a volume of 50 mL and centrifuged for 15 min at 1000×*g* and RT without brake. The PBMC layer was pipetted and transferred to a fresh 50 mL tube, where it was gently immersed in 50 mL E/B-PBS and centrifuged for 7 min at 400×*g* and RT with brake. After centrifugation the supernatant was aspirated and the cell pellet was immersed again in 50 mL E/B-PBS. This washing procedure was repeated trice, whereas after the first washing step, the cell pellet was immersed for 30 s in 2 mL sterile dH_2_O for the lysis of erythrocytes. Finally, the cell pellet was resuspended in 12 mL Minimum Essential Medium Eagle medium (MEM, Sigma Aldrich, Taufkirchen, Germany). The lymphocyte fraction was isolated from the cell suspension by centrifugation on a hyper-osmotic Percoll solution (19.4 mL Percoll (GE Healthcare, Freiburg, Germany), 16.6 mL dH_2_O, 4.0 mL 1.6 M NaCl solution). Therefore, 3 mL of the cell solution were overlaid onto 10 mL of the hyper-osmotic Percoll solution, centrifuged at 600×*g* and RT for 15 min with brake turned off. The PBMC including supernatant was separated from the lymphocyte cell pellet and washed in E/B-PBS by centrifugation at 400×*g*, RT for 7 min with brake. The cell pellet was then immersed in 3 mL MEM medium, added on an iso-osmotic Percoll solution (4.15 mL Percoll (GE Healthcare, Freiburg, Germany), 4.85 mL dH_2_O, 1.0 mL 1.5 M NaCl), and centrifuged for 15 min at 400×*g* and RT without brake. After a final washing step in E/B-PBS (7 min, 400×*g*, RT, with brake), the monocyte stock solution was prepared by suspending the cell pellet with MEM medium supplemented with 1% P/S and 2 mM l-glutamine (both Gibco®, Life Technologie, Darmstadt, Germany), 20 mM HEPES, and 10% FCS (both Sigma Aldrich, Taufkirchen, Germany) to a final concentration of 1·10^6^ monocytes/mL. A summary of the isolation procedure and the composition of solutions can be found in Table 3.

**Table 3:** Summary of the monocyte isolation process from buffy coats.

Monocyte isolation step	Procedure	Solutions
1st density gradient: Isolation of PBMC from buffy coats	Pancoll gradient centrifugation (15 min, 1000*g*, RT, without brake) of a 1:2 dilution of the buffy coat with E/B PBS	- Pancoll (1.077 g/L) - E/B PBS (PBS with 2 mM EDTA and 0.5% BSA)
Washing step (1x)	Immersion of PBMC layer in 50 mL E/B-PBS followed by centrifugation (7 min, 400*g*, RT, with brake)	- E/B-PBS
Erythrocyte lysis	Resuspension of cell pellet in 2 mL dH_2_O, 3sec	- Sterile dH_2_O
Washing step (2x)	Immersion of cell pellet in 50 mL E/B-PBS followed by centrifugation (7 min, 400*g*, RT, with brake)	- E/B-PBS
2nd density gradient: Separation of lymphocytes	hyperosmotic Percoll gradient centrifugation (15 min, 1000*g*, RT, no brake) of cell pellet immersed in 12 mL MEM medium, centrifugation of 3 mL cell suspension on 10 mL hyperosmotic solution	- hyperosmotic Percoll solution (19.4 mL Percoll, 16.6 mL dH_2_O, 4.0 mL 1.6 M NaCl) - MEM medium
Washing step (1x)	Separation of monocytes containing supernatant in 50 mL E/B-PBS followed by centrifugation (7 min, 400*g*, RT, with brake)	- E/B-PBS
3rd density gradient: isolation of monocytes	iso-osmotic Percoll gradient centrifugation (15 min, 400 *g,* RT, no brake) of cell pellet immersed in 3 mL MEM medium	- Iso-osmotic Percoll solution (4.15 mL Percoll, 4.85 mL dH_2_, 1.0 mL 1.5 M NaCl) - MEM medium
Washing step (1x)	Immersion of cell pellet in 50 mL E/B-PBS followed by centrifugation (7 min, 400*g*, RT)	- E/B-PBS
Preparation of monocyte solution	Immersion of cell pellet in 5 mL MEM medium	- MEM medium
Adjusting the working solution to 1·10^6^ monocytes/mL	Immersion of the calculated amount from the 5 mL MEM cell suspension into the corresponding amount of suppl. MEM medium	- Suppl. MEM medium

##### 2.5.1.2 Monocyte seeding and differentiation

One day before cell seeding, each ceramic scaffold was placed in a well of a 96-well plate and preconditioned in 200 μL MEM medium modified with 1% P/S at 37 °C in a 5% CO_2_ atmosphere. The medium was aspirated on the day of cell seeding and 6.6 × 10^5^ mononuclear cells per cm^2^ (corresponding to 2.0 cells per 96-well) in supplemented MEM medium were plated onto TMP-D and TMP-P scaffold, and on the reference material TCP. Poylsterene (PS) and glass (cover slips, Ø = 10 mm, Hartenstein GmbH, Würzburg, Germany) for FESEM investigations were used as established cell culture references. The cell number was halved on PS and glass compared to the culture on scaffolds. Glass cover slips were cultured in an 48-well plate. Non adherent cells were aspirated on the next day during medium exchange. Adherent mononuclear cells on different test surfaces were incubated in 200 μL (96-well) or 400 μL (48-well) differentiation medium, which was composed of MEM medium modified with 25 ng/mL human macrophage colony-stimulating factor (hM-CSF; Miltenyi Biotec, Bergisch Gladbach, Germany), 1% P/S, 20 mM HEPES (Sigma Adrich, Taufkirchen, Germany), 2 mM l-glutamine, 5% FCS, and 5% human serum (PAN-Biotech, Aidenbach, Germany). After 2 days, the differentiation medium was supplemented with 50 ng/mL human receptor activator of NF-κB ligand (RANKL, PerproTech, Hamburg, Germany). This medium initiated the differentiation of osteoclasts and thus this time point was defined as day 0 of osteoclast cell culture. The RANKL supplemented differentiation medium was exchanged every 3rd day until day 21.

#### 2.5.2 DNA quantification and cell activity

##### 2.5.2.1 Preparation of cell lysate

DNA and activity of intracellular enzymes were quantified by the analysis of lysed cells. For this purpose, cell scaffolds were washed with 200 μL PBS. Afterwards, the cell scaffolds were transferred to a fresh well plate, which allowed a separate lysis of the cells grown on the scaffolds and the cells grown on the well plate. Therefore, 300 μL lysis buffer were added to the scaffolds or the wells (1% Triton X-100 (Sigma Aldrich, Taufkirchen, Germany) in PBS). The cells were lysed on ice for 60 min, followed by 3 freeze-thaw cycles at −80 °C and room temperature.

##### 2.5.2.2 DNA quantification

The concentration of DNA in the lysed cells was quantified with the commercial Quant-iT PicoGreen Kit. In brief, 10 μL of each cell lysate and 190 μL of Quanti-iT PicoGreen working solution were added to a black 96-well plate and incubated in the dark for 5 min, at RT on a tilt shaker with 100 rpm. The fluorescence intensity was measured at an excitation wavelength of 485 nm and an emission wavelength of 535 nm using the Tecan plate reader. The DNA concentration was quantified according to a standard calibration curve.

##### 2.5.2.3 Enzyme activity and quantification

Intracellular carbonic anhydrase II (CA II) as well as extracellular tartrate resistant acid phosphatase (eTRAP) were investigated as osteoclast-specific marker proteins. The enzyme CA II catalyzes the acidification within the sealing zone between osteoclasts and bone, by which the demineralization of bone matrix is regulated [37]. Since CA II is strongly expressed in active osteoclasts, it was assayed as an early marker for osteoclast differentiation and resorption activity. TRAP, as a marker for increased bone turnover, is expressed by different cells in mammalians. Two isoforms of TRAP, i.e. 5a and 5b, are existing; the isoform 5b is considered to be released by osteoclasts during bone resorption and is used to display the active osteoclast-mediated resorption of bone or synthetic materials. The CA II activities were investigated at day 10, 16, and 21 and the eTRAP activities at day 9, 15, and 21 after the induction of osteoclastogenesis.

The quantification of the CA II activity is based on the hydrolysis of p-nitrophenyl acetate (pNPA) into p-nitrophenol (pNP) catalysed by CA II. 50 μL of each cell lysate were mixed with 50 μL of a CA II-substrate solution (12.5 mM tris(hydroxymethyl)-aminomethane, 75 mM NaCl, 2 mM pNPA (all from Sigma Aldrich, Taufkirchen, Germany)) followed by the measurement of absorption kinetics at 400 nm over a period of 30 min with a micro-plate reader. Solutions with different concentrations of pNP (Sigma Aldrich, Taufkirchen, Germany) in lysis buffer were used as calibration curve.

The quantification of eTRAP activity is based on the hydrolysis of p-nitrophenyl phosphate (pNPP) into pNP. The predominant detection of the isoform TRAP5b is possible by adjusting the pH of the reaction buffer to a value of 6.1, at which 5a is inactivated, while Type 5b remains highly active [38]. For this purpose, measurements were conducted by mixing 50 μL of supernatants with the 150 μL TRAP reaction buffer (100 mM sodium acetate (Merck KGaA, Darmstadt, Germany), 50 mM sodium tartrate (Carl Roth GmbH + Co. KG Karlsruhe, Germany), and 7.5 mM pNPP (Sigma Aldrich, Taufkirchen, Germany), pH = 6.1). The supernatants were collected 1 day before cell lysis. The solutions were incubated for 60 min at 37 °C under 5% CO_2_ atmosphere, after which the enzymatic reaction was terminated by the addition of 50 μL 0.3 M NaOH. The conversion to pNP was determined by absorption measurement at an excitation wavelength of 405 nm and an emission wavelength of 620 nm using a micro plate reader, whereas the optical density was correlated to pNP standards.

#### 2.5.3 Fluorescence and histochemical tartrate resistant acid phosphatase staining of osteoclasts

##### 2.5.3.1 Fluorescence staining

Osteoclast differentiation was analyzed using immunoﬂuorescence microscopy. On days 10, 16, and 22 after cell seeding the phenotypes of the cells grown on the scaffolds and on the well plate were analyzed separately. Therefore, the cells on the scaffolds were rinsed with PBS, fixed in ice-cold Roti®-Histofix (Carl Roth GmbH + Co. KG, Karlsruhe, Germany) for 5 min, and permeabilized with 0.2% Triton X-100 in PBS for 5 min at RT after having been rinsed twice with PBS. Thereafter, unspecific binding sites on the scaffold were blocked by incubation in 2.5% BSA (100 μL/well, Sigma Aldrich, Taufkirchen, Germany) in PBS for 30 min at RT. Cytoskeletal F-actin was labelled with Phalloidin-iFluor 555 reagent (Abcam, Berlin, Germany) for 60 min in the dark using 200 μL/well of a 0.1% phalloidin solution. After 50 min 20 μL of a 133 μg/mL Hoechst 33342 solution (Thermo Fisher Scientific Inc., Waltham, MA, USA) were added to each well for nuclei counter staining. After washing with PBS, the scaffolds were retrieved from the well and stored in the wells of a fresh plate. Cells grown on scaffolds and cells grown on the culture plate were stored in 200 μL PBS until microscopic investigation. Imaging was performed with a fluorescence microscope Axio Observer (Carl Zeiss AG, Oberkochen, Germany) at 556 nm excitation wavelength and 574 nm emission wavelength.

##### 2.5.3.2 Histochemical tartrate resistant acid phosphatase staining

Tartrate resistant acid phosphatase (TRAP) staining was performed using a commercially available acid phosphatase kit from Sigma-Aldrich (Cat No. 387, Sigma Aldrich, Schnelldorf, Germany). The staining was performed according to the manufacturer's instructions. In detail, after aspiration of the culture medium, the cells were fixed with 100 μL fixative solution (3.316 mL acetone, 1.276 mL citrate solution (Sigma Aldrich, Schnelldorf, Germany), and 0.408 mL 37% formaldehyde) for 30 s. Thereafter the samples were washed twice with 200 μL PBS. Then, 100 μL of a freshly prepared diazotized Fast Garnet GBC Base Naphthol AS-BI phosphoric acid staining solution (Sigma Aldrich, Schnelldorf, Germany) was added to the wells followed by incubation at 37 °C and 5% CO_2_. After 60 min the staining solution was aspirated, the surfaces were washed twice with 200 μL dH_2_O and dried overnight at RT. The transmitted light microscope VisiScope IT 404 (VWR International, Leuven, Belgium) and the software ZEN 2.3 (blue edition) from Carl Zeiss Microscopy GmbH (Zeiss, Oberkochen, Germany) were used to analyze the PS reference. The scaffolds were analyzed with the SteREO Discovery.V20 stereomicroscope and the ZEN 2012 (blue edition) software (both Zeiss GmbH, Oberkochen, Germany).

#### 2.5.4 Scanning electron microscopy of cell seeded scaffolds

FESEM (Crossbeam CB 340, Zeiss, Oberkochen, Germany) was used to investigate the microstructure of the scaffolds, osteoclast morphology as well as resorption pits on day 17 of osteoclast culture. For reference purposes, osteoclasts were cultured on glass. For the analysis of resorption lacunae, cells were detached from the ceramic surface by immersion in 1% Triton X-100 in PBS. The cell lysis procedure was followed by washing of the scaffolds in deionized water and by drying at 60 °C for 12 h. For the analysis of cell morphology, osteoclasts on scaffolds and on glass were fixed with 6% glutaraldehyde, dehydrated in an ascending acetone series at RT followed by critical point drying (Critical Point Dryer CPD 030, Bal-Tec, Witten, Germany). Afterwards, all scaffolds or glass samples were sputter-coated with platinum and analyzed by FESEM.

### 2.6 Bioresorption of ceramics

The calculation of chemical erosion and cell-mediated resorption was based on the measurement of ion concentration, in particular Mg^2+^, Ca^2+^, and PO_4_^3−^, in the supernatants over a period of 3 weeks. For the observation of chemical erosion, ceramic discs were soaked in 200 μL supplemented MEM medium without cells and growth factor under cell culture conditions. Like the scaffolds of the *in vitro* study, the scaffolds of the biodegradation study were conditioned in cell media 2 days before the start of the study. The medium was refreshed every 3 days. The weight loss was calculated by weighing the air dried samples before and after the degradation study. Supernatants from the osteoclast cell culture study and the chemical degradation study were harvested and the contents of Mg^2+^, Ca^2+^, PO_4_^3−^ ions were determined by means of inductively coupled plasma mass spectrometry (ICP-MS, *Thermo Scientific™ iCAP™ RQ*, Thermo Fisher, Darmstadt, Germany). Therefore, the media were centrifuged at 1000×*g* in order to remove cement and cell debris. Thereafter, a 1:80 dilution in 0.69% nitric acid was prepared for each supernatant and ion composition was determined. The osteoclast-mediated resorption of the ceramic was calculated by subtracting the ion content released from scaffolds immersed in medium without cells from the total ion release observed during osteoclast culture. Moreover, the pH value of the supernatants was determined during osteoclast culture on scaffolds with a pH meter WTW inoLab® pH 730 (Xylem Analytics Germany Sales GmbH & Co. KG, Weilheim, Germany).

### 2.7 Statistical methods

Each experimental set up comprised a sample size of *n* = 3, unless it is not mentioned otherwise. The statistical analysis between different scaffolds was performed using the Two-Way ANOVA and Tukey-test (Origin 7G, OriginLab Corporation, USA). Values of p < 0.05 were considered as statistically significant. All results were expressed as mean ± standard deviation.

## 3 Results and discussion

### 3.1 Surface morphology, porosity, and phase composition of the scaffolds

After printing the scaffolds consisted of ceramic particles and inter-particular swollen cellulose. The cellulose was eliminated during the sintering process, so that the final scaffolds consisted only of the pure ceramic components, namely pure farringtonite (TMP-Sin) or a mixture of 12 wt% α-TCP and 88 wt% β-TCP (Fig. 1a–d). The phase composition of the TMP-Sin scaffolds was changed by the treatment with either an acidic or an alkaline solution. Here, TMP-Sin ceramic was partially dissolved in an ammonium phosphate solution or in phosphoric acid, which resulted in the precipitation of 24 wt% struvite (NH_4_MgPO_4_ · 6 H_2_O, eq. (1)) or 43 wt% newberyite (MgHPO_4_ · 3 H_2_O, eq. (2)) (Fig. 1c).

(1) 2 Mg_3_(PO_4_)_2_ + 3 (NH_4_)_2_HPO_4_ + 36 H_2_O → 6 NH_4_MgPO_4_ · 6 H_2_O + H_3_PO_4_

(2) Mg_3_(PO_4_)_2_ + H_3_PO_4_ + 9 H_2_O → 3 MgHPO_4_ · 3 H_2_O

**Fig. 1:**
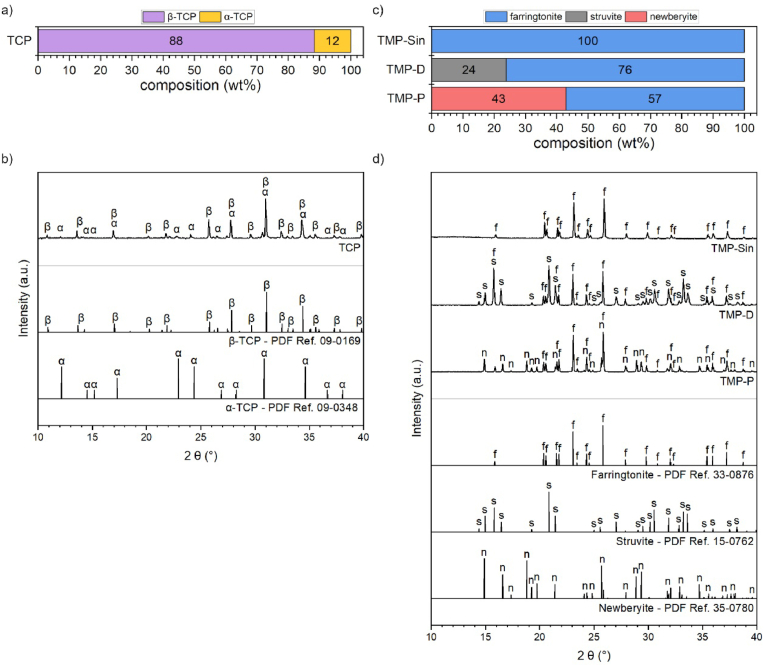
a-b) Chemical composition of printed and sintered TCP scaffolds. c-d) chemical composition of printed and sintered scaffolds (TMP-Sin) and after alkaline (TMP-D) or acid (TMP-P) treatment. α-TCP (α), β-TCP (β), farringtonite (f), newberyite (n), struvite (s).

The conversion to a highly hydrated MP mineral phase increases the solubility under physiological conditions, which was already verified *in vivo* for struvite-containing bone cements [30]. In this special case, the solubility of the ceramic scaffolds increased from initially 2.15 mg/L (TMP-Sin) by the conversion to struvite (solubility >8.3 mg/L) or newberyite (>1.69·10^3^ mg/L) [29]. All TMP-Sin reaction products showed a higher solubility, as compared to α-TCP (2.5 mg/L) or β-TCP (0.5 mg/L) [12].

All scaffolds were composed of fused ceramic particles, that formed a porous and rough network (Fig. 2), which was described as characteristic for powder printed ceramics [4,39]. Among the investigated materials, TCP scaffolds showed the highest interconnected porosity of (46.4 ± 7.3) % and modal pore size of (58 ± 7) μm. The ceramic powder particles were fused to interconnected agglomerates with a diameter of (105 ± 35) μm during sintering and still showed a texture on the microscale of (18 ± 5) μm resulting from the initial size of particles. As a consequence of newberyite formation caused by acid treatment, TMP-P scaffold had a slightly reduced porosity and modal pore size of (35.7 ± 5.2) % and (29 ± 4) μm, respectively. However, the surface topography showed a marked similarity to TCP scaffolds. The ceramic network of TMP-P was composed of agglomerates of (61 ± 11) μm in size, which were structured on the scale of (22 ± 4) μm. On a microstructural point of view, TMP-D differed immensely from the other scaffolds. The TMP-D surface was characterized by prismatic particles with lengths >100 μm. Although these particles were imbedded in a relatively dense matrix with a total open porosity of (14.8 ± 1.6) % and a modal pore size of (14 ± 3) μm, the wide variation of particle forms and shapes resulted in a coarse-grained surface and partly large distances between particles of a few 100 μm.

**Fig. 2:**
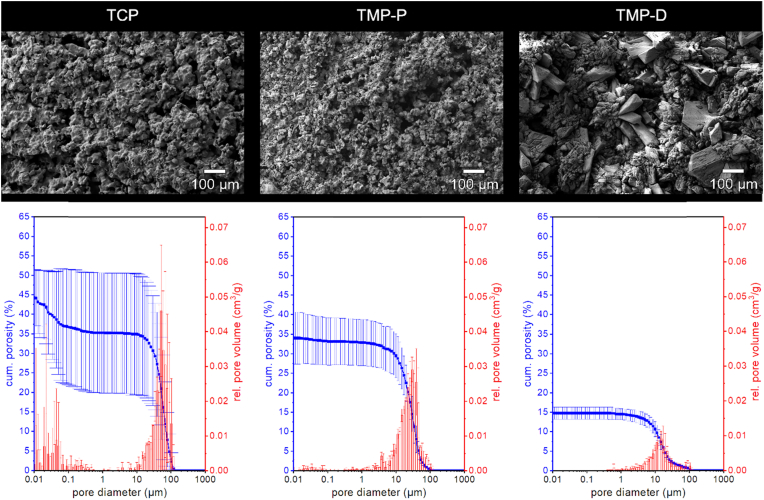
Surface morphology, porosity, and pore size distribution of TCP, TMP-P, and TMP-D scaffolds.

### 3.2 Bioactivity

It is scientific consensus that the bone bonding ability of bone replacement materials *in vivo* can be estimated *in vitro* by the determination of apatite precipitation on a biomaterial, which has been immersed in a serum-like solution under physiological conditions [10]. Biomaterials, which accelerate the formation of a bone-like HA layer under these conditions, have been defined as bioactive [10] and can potentially induce intrinsic osteoinduction *in vivo* [40]. Thus, *bioactivity* testing can be used to interpret the bone bonding capacity of biomaterials observed *in vitro* or *in vivo*. Bohner et al. pointed out that apatite formation is not inevitably associated with new bone formation [10], but it has to be recognized as a pre-requisite, as several studies confirmed the precipitation of an apatite layer prior to bone formation *in vivo* [[40], [41], [42]]. In general, the formation of biomimetic apatite is induced, if in serum-like solutions, which are supersaturated with respect to calcium and phosphate, the pH levels or the concentrations of the ionic components of poorly soluble apatites are increased and/or apatite nucleation sites are provided [10]. In our study, we could confirm the *bioactivity* of clinically established TCP by a superficial formation of desert-rose like crystals (Fig. 3a), which is the typical morphology for HA with low or no carbonate content precipitated in SBF [43]. The plate-like crystals grew on spongy (Fig. 3b, *) or spherical (Fig. 3b, o) substructures.

**Fig. 3:**
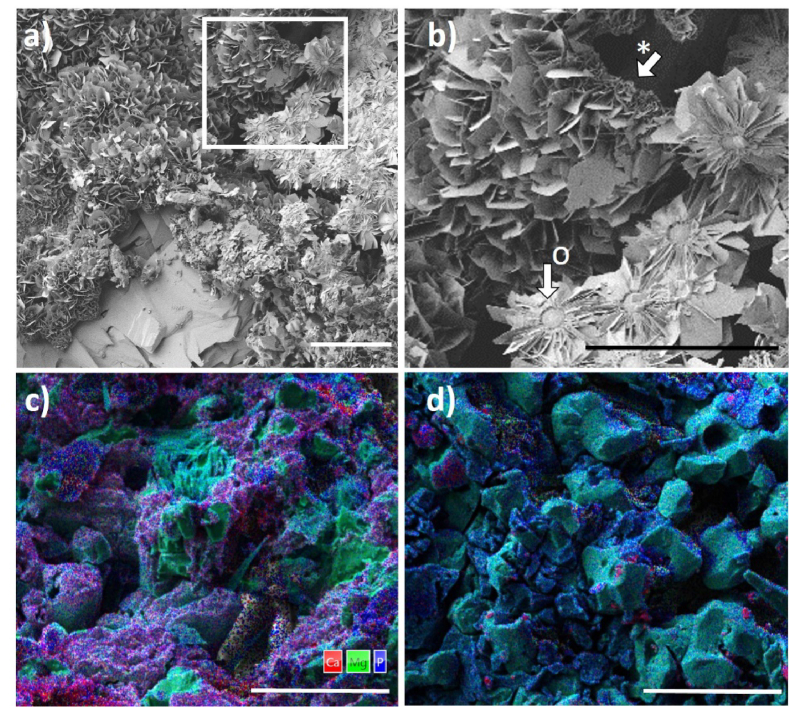
a) Microstructure of precipitated HA on TCP after immersion in SBF for 7 days. b) HA precipitates had a plate like structure (*) and grew on spherical substructures (o). Microstructure and local ion composition of TMP-D (c) and TMP-P (d) scaffolds after immersion in SBF for 7 days. Scale bars = 50 μm. Ca: red, Mg: green, P: blue. (For interpretation of the references to colour in this figure legend, the reader is referred to the Web version of this article.)

A precipitation of globular closely packed crystals, the typical morphology of amorphous or low crystalline CHA [[43], [44], [45]], was observed on TMP-D and TMP-P by means of FESEM and EDX analysis (Fig. 2c–d and suppl. Fig. S1). Due to the reduced crystallinity and content, the precipitated HA could not reliably identified by XRD (suppl. Fig. S2). Nevertheless, Mg^2+^ is known to inhibit apatite crystallization and to stabilize the formation of amorphous CHA. This phenomenon was also confirmed by a study of Meininger et al. [46]. Here, the authors observed, that during immersion of a struvite coated titanium disc, the thin MP coating was completely dissolved and converted into low-crystalline HA.

It has to be pointed out, that CHA and in particular amorphous CHA are characterized by an increased solubility and bioavailability [47,48]. The ion distribution detected by EDX confirmed the precipitation of a CHA layer on TMP-D, since Mg^2+^ was concentrated in the bulk material, while Ca^2+^ and PO_4_^3−^ were predominantly found in a thin superficial layer. The Ca^2+^ content in the CHA precipitate was higher in TMP-D compared to TMP-P.

### 3.3 Cytocompatibility

**Fig. 4:**
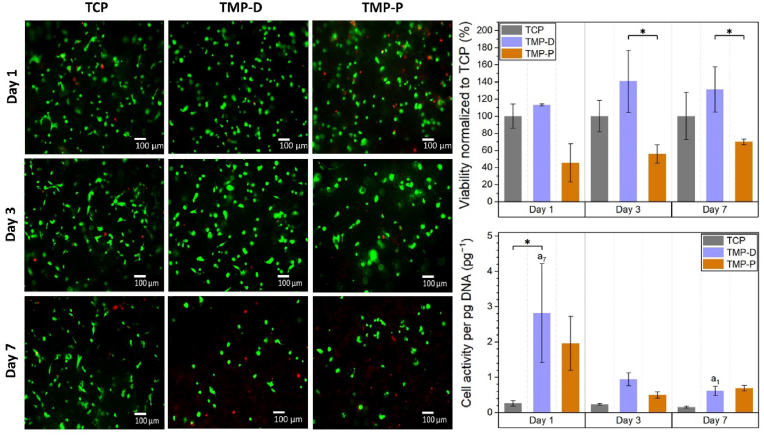
Cytotoxicity of TMP scaffolds was evaluated by live/dead staining and determination of cell viability and activity of human osteoblast (hFOB 1.19) cultured on TCP (material reference), TMP-D, and TMP-P scaffolds. Significant differences (p < 0.05) at a specific time point between different material groups are labelled with * and significant differences within one material group at consecutive time points are labelled with “a” with an index defining the time point of comparison.

The cytocompatibility of MP based scaffold materials was evaluated by means of cell attachment, viability, and activity. Live/dead staining showed that cells of the human fetal osteoblastic cell line hFOB 1.19 attached to all ceramic surfaces (Fig. 4). It was noticed that the cells maintained a rounded shape throughout the cultivation period, which is attributed to the irregular culture surface provided by the powder printed scaffolds. During this time, most of the cells in all 3 groups, but at least 79% of the total cell population, stayed vital (green). The percentage of viable cells on day 1, 3, and 7 were (79 ± 12) %, (91 ± 5) %, (63 ± 17) % for TCP, (87 ± 10) %, (78 ± 14) %, (56 ± 23) % for TMP-D, and (87 ± 10) %, (70 ± 22) %, (85 ± 19) % for TMP-P. None of the investigated scaffold materials prevented cell proliferation. However, within the first 2 days cell growth on TCP was inhibited compared to the expected doubling of hFOB cells of 36 h [49]. Instead, doubling time of cells cultured on TMP-D and TMP-P was reduced to 48 h and 24 h, respectively. This was in accordance with the increased metabolic activity observed for cells cultured on both MP scaffold materials (Fig. 4). Under the aspect of viability, TCP and TMP-D caused only slight cytotoxic side effects, while TMP-P showed mild to moderate cytocompatibility (Fig. 4) [35].

### 3.4 Osteoclast attachment

**Fig. 5:**
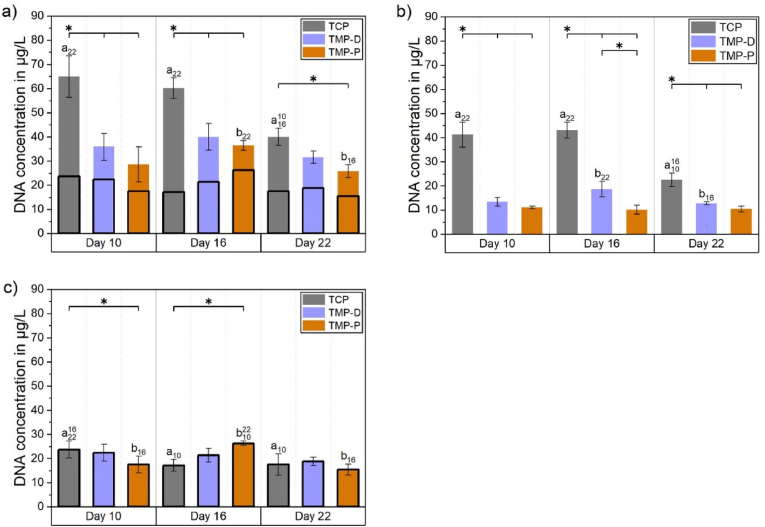
a) Total DNA content divided in cells attached to the well (bottom part of the bar) or to the scaffold (upper part of the bar) 10, 16, and 22 days after induction of osteoclast differentiation by the addition of the growth factors hM-CSF and RANKL. The total DNA amount in the culture well (a) was separated in (b) DNA amount on the scaffold and (c) DNA amount on the well bottom. Significant differences (p < 0.05) between different materials at one time point are labelled with *, while significant differences (p < 0.05) between cell populations grew at the same scaffold but at different time point are marked with letters “a” (TCP) or “b” (TMP). Initials indicating the time points, which were compared.

Although DNA quantification is no reliable indicator for the number of osteoclasts, it has been used to estimate the cytocompatibility based on measured cell adhesion. In general, 10–20% of the initially seeded monocytes (2·10^5^ cells or ∼400 μg/L DNA for scaffolds, 1·10^5^ cells for PS) adhered on pure well plates (PS) or on the scaffold surfaces. In particular, 15–20% or 2–10% of the monocytes became adherent to PS or to the scaffolds, respectively (suppl. Fig. S3). The DNA amount was significantly decreasing over time for TCP, which was in accordance with the facts that differentiation of osteoclast does not result in DNA increase and undifferentiated mononuclear cells might be washed off from the surfaces during cell cultivation (Fig. 5). The highest DNA content was observed for TCP, whereby cell attachment was significantly higher compared to TMP scaffolds at each time point (Fig. 5a and b). The overall DNA content on TMP was always higher than 44% (TMP-P, day 10) of the DNA content detected on TCP at the same time point. It is striking, that between day 10 and 16 no significant decrease with respect to the DNA quantity over time was observed for all TMP scaffolds (Fig. 5b), which indicated a stable cell population without cell detachment (Fig. 5b and c).

**Fig. 6:**
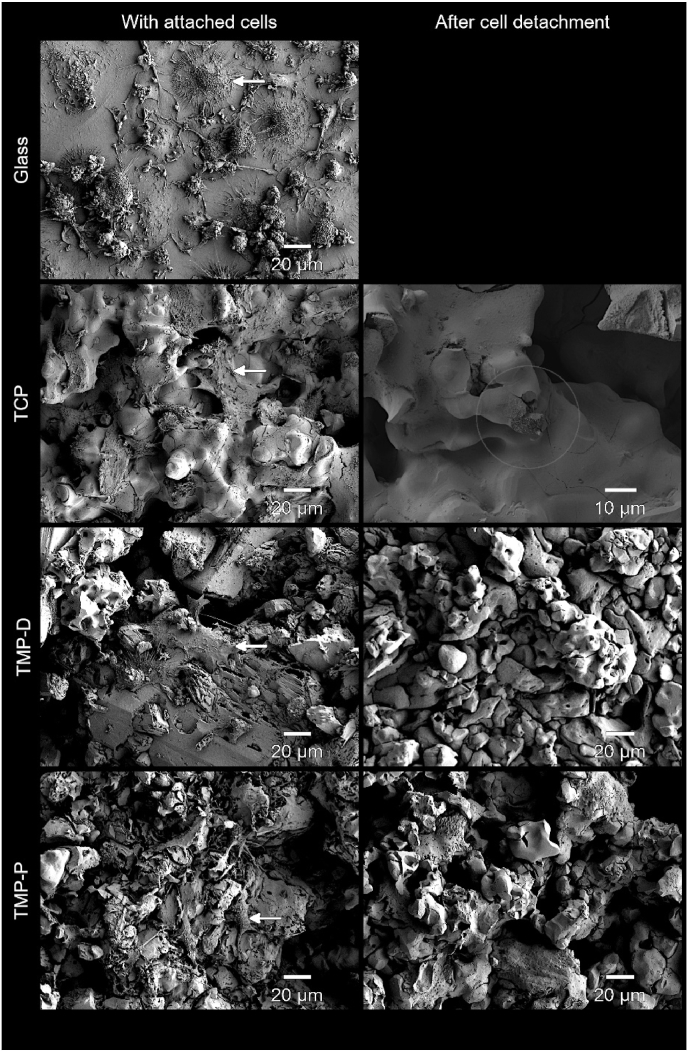
FESEM images at day 17 after initiation of osteoclast differentiation in the cell culture with RANKL showed the growth of osteoclasts (white arrows). Osteoclast-like giant cells were found on all ceramic surfaces. After cell detachment after 17 days cellular resorption pits were detected for TCP exclusively (circled area). Glass was used as established control for cell culture.

Cell adhesion was also confirmed by FESEM imaging (Fig. 6). The largest cell size was detected on the culture control material glass, whereat 26% of the cell population had cell diameters of >50 μm (Fig. 6). These cells showed the characteristic morphology of mature osteoclasts, i.e. ovoid shaped cells with a dome-like centre structure, tightly attached to the surface [50]. In comparison, cells on TCP, TMP-D, and TMP-P showed a smaller diameter of 20–40 μm. This can be attributed to the irregular and porous microstructure of the ceramic (Fig. 2), which limited cell fusion and prevented cells to form an osteoclast-specific morphology (Fig. 6). Instead, the cells spanned the intra-crystalline distances by spreading up to 30 μm long lamellipodia. Typical resorption pits were exclusively found on TCP scaffolds, and no evidence of resorption could be identified for TMPs (Fig. 6). Besides this, all detected cells showed attachment to the scaffolds.

### 3.5 Cell staining and osteoclast differentiation

The fusion of isolated human monocytes to multinucleated preosteoclasts and the differentiation to mature osteoclasts was analyzed by the visualization of F-actin ring formation and cell morphology, as well as by TRAP staining.

**Fig. 7:**
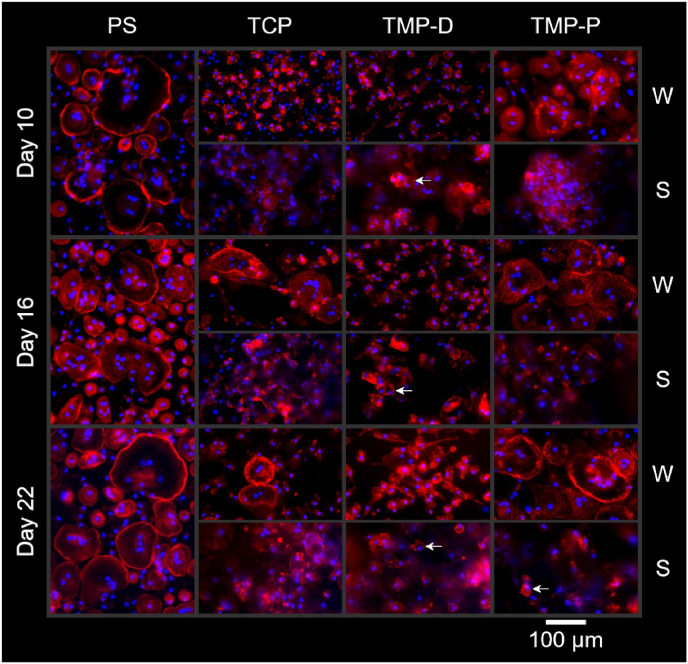
Fluorescence staining of cells cultured on the cell culture control polystyrene (PS) and on ceramic scaffolds (TCP and TMP-D/-P). Cells, which grew on the well plate bottom (W) and on the scaffold (S) were analyzed separately. F-actin is stained in red, cell nuclei are stained in blue. Cells showing actin ring formation, a minimum of 3 nuclei, and a cell size >50 μm were classified as active osteoclasts. These mature osteoclasts could be verified from day 10 onwards for PS, for TCP at day 16 and 22, and for TMP-D and –P for day 22. Mononuclear osteoclasts were detected on TMP-D after 16 days and on TMP-P after 22 days (labelled with an arrow). (For interpretation of the references to colour in this figure legend, the reader is referred to the Web version of this article.)

The fluorescence staining of F-actin and cell nuclei confirmed the formation of multinucleated cells (more than 3 nuclei per cell) on the control (PS) and the reference material (TCP) as well as on TMP-P (Fig. 7). The irregular topography of ceramics prevented an accurate focusing of whole cells and thus the visualization of eventually formed sealing zones. This difficulty was also observed in other studies, which investigated the bioresorption of ceramics by direct cultivation of osteoclasts on scaffolds [48,51]. However, in order to evaluate the influence of different scaffold materials on osteoclasts, cells which were grown on scaffolds (Fig. 7 (S)) and on tissue culture plastic beneath the scaffolds in the same well (Fig. 7 (W)) were imaged separately. It should be noted, that cells attached to the bottom of the well plates were not in direct contact with the scaffolds, but their cell behavior was influenced by the dissolved scaffold components in the culture medium. In general, chemical degradation of the scaffold material affects the ionic composition and pH of the medium. The influence of the soluble scaffold components on the cells is usually investigated in so-called indirect cell culture assays, in which the cells are cultured in the eluates of the materials. Since the pH values during cell culture did not deviate significantly from the pH value of the pure culture medium (pH = 7.5), no influence on cell behavior can be assumed. Between day 9 and day 21 pH values varied between 7.32 and 7.44 for TCP, 7.40–7.45 for TMP-D, and 7.48–7.55 for TMP-P (suppl. Fig. S4).

It was found that the time point as well as the extent of cell fusion and osteoclast formation differed, depending on the scaffold material. While after 10 days mature osteoclasts (more than 3 nuclei, cell size above 50 μm, actin ring formation) were observed on the PS control, the osteoclastogenesis was delayed for TCP and TMP (Fig. 7 (W)). At this time, preosteoclasts containing 2-3 nuclei were formed on both TMP or the respective wells and osteoclastogenesis on TCP was in the early phase of monocyte cluster formation. With advancing culture time, the number of mature multinuclear osteoclasts with fully formed actin sealing zones increased for cells grown on well plates of TCP (>16 days) and TMP-P culture (>10 days). It is striking that throughout the investigation period cell populations on TMP-D scaffolds or the respective well bottoms were dominated by mono- and binuclear cells with a diameter of 52–79 μm. These cells could be categorized to either preosteoclasts or mononuclear osteoclasts (Fig. 7 TMP-D, labelled with an arrow) with actin rings. The actin organization in these cells changed from a homogenous intracellular distribution on day 10 to randomly distributed intracellular podosome clusters on day 16, characteristic for preosteoclasts [24,52]. Nevertheless, the fluorescence staining provided no indication for the formation of mature multinuclear osteoclasts on TMP-D until the end of the study. The existence of the characteristic peripheral actin belt could be proven only for osteoclasts grown directly on the well plates or for mononuclear cells on TMP-D and TMP-P. Although it was described that resorption-active osteoclasts on apatite-containing matrices built intracellular actin rings [52], the visualization of actin rings was either not possible on the scaffolds or was only successful for mononuclear cells. The difficulty to distinguish the differentiation stages of osteoclasts for cells grown on rough ceramics by fluorescence staining (Fig. 7 (S)) could be partially overcome by TRAP staining.

**Fig. 8:**
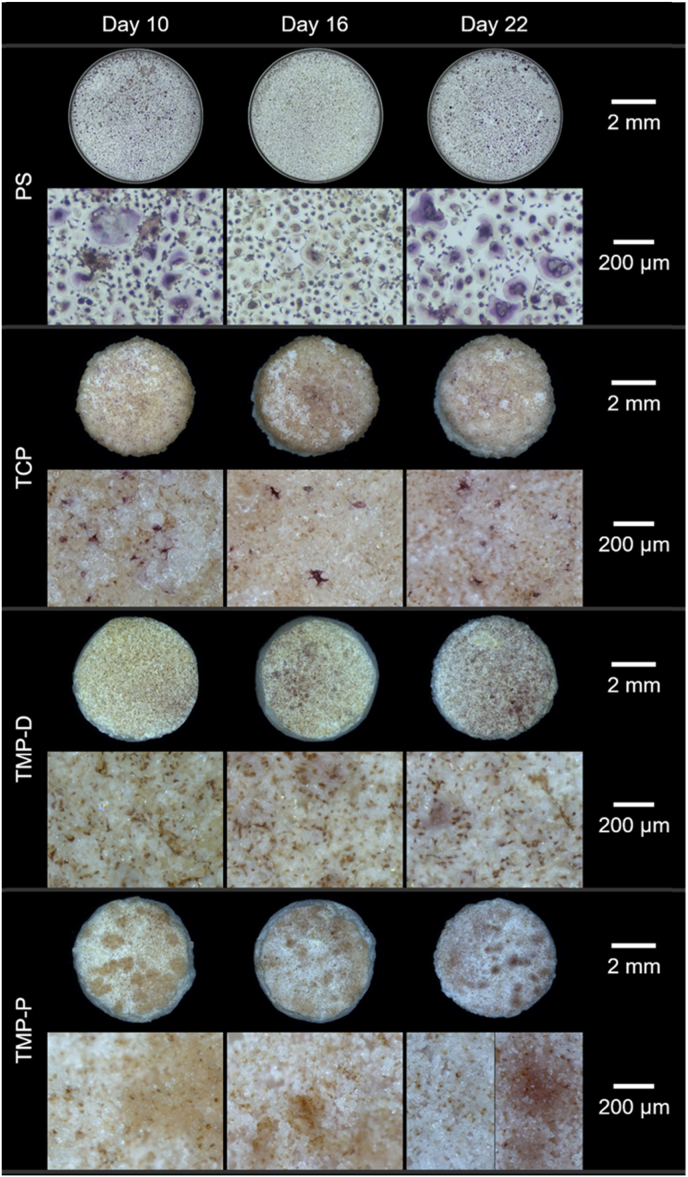
Overview of TRAP-stained monocytes-derived human (pre)osteoclasts on the cell culture control polystyrene (PS) and scaffolds at day 10, 16, and 22. Maroon or purple staining indicates the presence of TRAP-active cells, yellowish areas indicate TRAP-negative cells. TRAP + cells could be detected on each material, whereas cells with a size >50 μm are categorized as mature osteoclasts and were visible on PS (day 10–22) and TMP-P (day 22). TRAP + preosteoclasts or mononuclear osteoclasts with a cell size 25–50 μm could be detected on TMP-D and TCP. Upper rows show the complete culture surface (PS or ceramic scaffold), while lower rows show a 80× magnification of the culture surface. (For interpretation of the references to colour in this figure legend, the reader is referred to the Web version of this article.)

It was already described that besides mature osteoclasts also osteoclast precursors, mononuclear osteoclasts, and macrophages can be TRAP-positive (TRAP+) and will be stained in red colour of different intensities [53,54]. TRAP + cells could be detected on PS and on TCP on days 10–22 and on TMP on days 16 and 22 (Fig. 8). The largest number of TRAP + cells was detected on PS, followed by TMP-D, TCP, and TMP-P. It is striking, that only TRAP + cells with a cell size between 25 and 50 μm could be visualized for TCP and TMP-D. These cells were classified as preosteoclasts or mononuclear osteoclasts due to their size and limited number of nuclei detected by fluorescence staining. Based on the cell size of above 50 μm, TRAP + cells on PS from day 10 and on TMP-P on day 22 could be assigned to mature multinuclear osteoclasts. Our results indicated an inhibition of osteoclastogenesis for monocytes cultured in presence of TMP-D, a delay for cells cultured with TCP, and an early onset of osteoclast differentiation for TMP-P. Furthermore, again no cytotoxic effects could be observed for any tested material.

The existence of the characteristic peripheral actin rings could be proven only for osteoclasts grown directly on the well plates or for mononuclear cells on TMP-D and TMP-P. Although it was described that resorption-active osteoclasts on apatite-containing matrices built intracellular actin rings [55], the visualization of actin rings was either not possible on the scaffolds or was only successful for mononuclear cells. In summary, the findings were in accordance with a study of Ciapetti et al., who described a strong correlation of scaffold topography and osteoclast differentiation [56]. The authors have shown that the fusion of precursor cells, the maturation of osteoclasts as well as the osteoclast activity were reduced on HA scaffolds, when surface irregularities increased on the micro scale, which can be attributed to impeded cell movement [57]. The irregular structure of the ceramics in the study at hand can be regarded as a cause of prevented cell-cell fusion and osteoclast migration. However, it can be emphasized that osteoclast formation on the well plate near or under the scaffold was either not affected (TMP-P) or cell-cell fusion was reduced (TMP-D). Accordingly, our data suggest that osteoclastgenesis and bone regeneration, especially in the implant periphery, will either not be affected or even tissue regeneration *in vivo* will be increased due to downregulation of the formation of multinucleated and highly resorption active osteoclasts [58]. This assumption is strengthened by a study of Lee et al., which showed an increased bone formation as a result of inhibited osteoclast fusion and maturation in mice [57]; a consequence of the positive correlation between the number of osteoclast nuclei and the number of osteoblast as well as the activating properties of osteoclasts on osteoblasts [58,59].

### 3.6 Cell activities

**Fig. 9:**
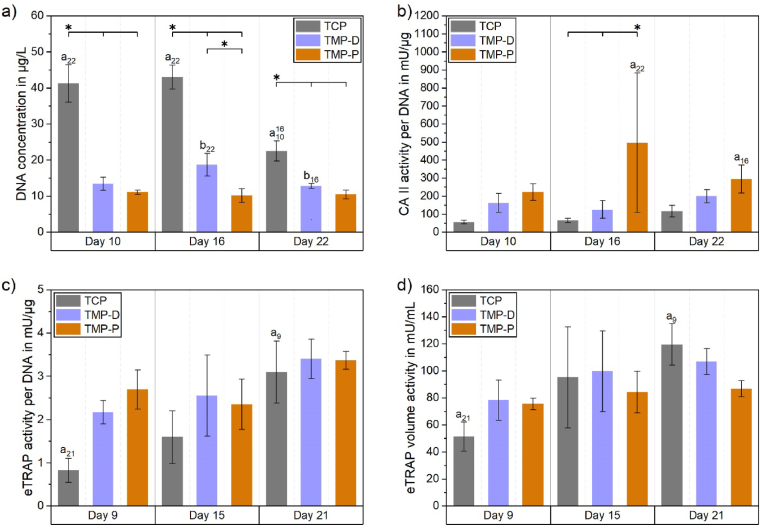
a) DNA content on the scaffolds and CA II (b) and eTRAP (c–d) activities of the cells. CA II activity (b) was determined for cells grown on the scaffolds, while eTRAP (c/d) was determined in the culture supernatants and display TRAP released from cells grown on the scaffolds and on the well plates. Significant differences (p < 0.05) within one material group at consecutive time points are labelled with “a” or “b” and time point initials and significant differences at a specific time point between different material groups are labelled with *.

Besides staining procedures, the resorption activity of osteoclasts can be verified by the quantification of specific enzymes, e.g. TRAP and CA II. The membrane-bound enzyme CA II is expressed in mature osteoclasts [37,60] and is vital for bone resorption as it catalyzes the production of protons. As such, CA II can be used as a marker for resorption-active cells. In the following, only osteoclasts adhered to the scaffolds were included in the analysis (Fig. 9a). We could prove a more than 2-times higher CA II activity per DNA for cells grown on TMP, as compared to cells attached to the reference TCP, at each time point (Fig. 9b). This increase was even significant in the case of TMP-P. Interestingly, a similar upregulation of CA II and secreted TRAP was only found for native tissues, collagen gels [61] or dentin [58], which emphasized the cell compatibility of TMP and verified the differentiation to resorption-active osteoclasts on both TMP based materials.

A similar trend was observed for eTRAP activity (Fig. 9c). As compared to cells cultured on TCP, the TRAP secretion per DNA was 2-3-fold upregulated for TMP on day 9, followed by a further slight increase of 20% and 36% for TMP-P and TMP-D up to day 21, respectively. A 3.7-fold upregulation for cells cultured under the influence of TCP resulted in a nearly equal level of eTRAP secretion per DNA (Fig. 9c) for all tested materials between day 15 and 21. A similar trend was observed for the total volume eTRAP activity (Fig. 9d). Here, the TRAP secretion of cells on TCP was nearly doubled from day 9 (51 mU/mL) up to day 15 (95 mU/mL), and increased by further 25% (119 mU/mL) on day 21. Meanwhile, cells on TMP-D showed a low rising trend (day 9: 78 mU/mL, day 15: 100 mU/mL, day 21: 107 mU/mL). The increase in eTRAP activity indicated that differentiation of preosteoclasts had not yet been completed until day 15 for TMP-D or day 21 for TCP. Generally, the differences in eTRAP acitivity of the cells cultured on different materials at a specific were not significant at any time point. Furthermore, there was no correlation between the amount of secreted eTRAP and the average number of nuclei in the osteoclasts. The stagnating eTRAP value for TMP-P (day 9: 76 mU/mL, day 15: 84 mU/mL, day 21: 87 mU/mL) indicated that the osteoclast maturation had already finished on day 9. Together with the results of fluorescence staining, the results of eTRAP and CA II activity analysis confirmed an early start of osteoclast differentiation for TMP-based materials.

These results confirm the statement, that CA II activity reflects the resorption activity of cells on a ceramic material. Since this has so far only been shown for calcium phosphates, a generalization to ceramic materials seems now conclusive.

### 3.7 Chemical degradation and cellular resorption

**Fig. 10:**
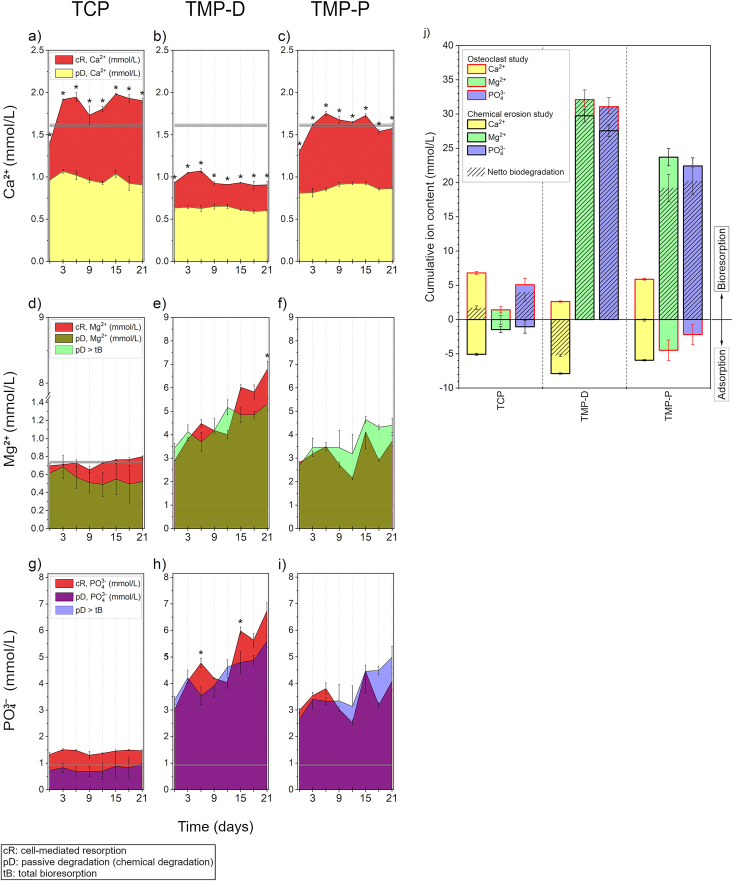
Adsorption and degradation kinetic of Ca^2+^ (a–c), Mg^2+^ (d–f), and PO_4_^3−^ (g–i) ions during immersion of TCP (material reference) (a, d, g), TMP-D (b, e, h), and TMP-P (c, f, i). The figures show the ion concentration in supernatants of scaffolds cultured in cell media with or without cells. j) The cumulative ion concentrations showed, that Ca^2+^ was precipitated (area <0) followed by cell-mediated resorption for each scaffold material. In the case of TCP the Mg^2+^-doped HA layer and partially the scaffold material were resorbed by osteoclast (dashed areas >0). Ca^2+^ precipitates were partially or complete resorbed by cells for TMP-D and TMP-P, respectively. The release of Mg^2+^ and PO_4_^3−^ during cell culture were identical or less compared to cell-free immersion of TMP-D or TMP-P, which indicated chemical degradation of the material. Significant differences between ion concentrations in supernatants of cell-free and cell-seeded scaffolds are labelled with * (p < 0.05).

The chemical degradation and the cell-mediated resorption of ceramic scaffolds were evaluated by weighing and by the determination of the ionic composition of the supernatants of the immersed samples. During the incubation of the cell-free scaffolds in culture media all ceramic surfaces induced calcium adsorption (Fig. 10 a - c) in the form of CHA precipitation, which was in accordance with the observation made during the bioactivity measurement and EDX analysis (Fig. 3). TMP-D showed the highest absolute Ca^2+^ precipitation (7.9 ± 0.1 mmol/L, approx. 506 ± 6 μg/scaffold), followed by TMP-P (5.9 ± 0.1 mmol/L, approx. 381 ± 7 μg/scaffold), and TCP (5.1 ± 0.2 mmol/L, approx. 326 ± 10 μg/scaffold) (Fig. 10j). Thus, the calcium levels in the supernatants of the cell-free scaffolds were reduced, as compared to the initial Ca^2+^ level of the culture medium (1.6 mmol/L, grey line in Fig. 10a–c) as well as to the physiological Ca^2+^ level found in serum: 1.16–1.31 mmol/L. In particular, in the case of TMP Ca^2+^-concentrations were similar to the one found during new bone formation (0.5 mmol/L) [62]. Such low Ca^2+^ levels are known to initiate osteoclast adhesion and to promote osteoclast formation [63]. The high binding affinity of osteoclasts under hypophysiological conditions resulted in a favoured attachment of cells to all material variants and a constant amount of DNA for TMP over 22 days. This also resulted into a cell-mediated continuous resorption of the CHA layer with a rate of 8 μg Ca^2+^/scaffold/day for TMP-D and 18 μg Ca^2+^/scaffold/day for TMP-P. In the case of TCP, the Ca^2+^ release significantly exceeded the total Ca^2+^ adsorption, demonstrating an active cellular resorption of the TCP ceramic (red area Fig. 10a and dashed area 10j) with a rate of 21 μg Ca^2+^/scaffold/day. These resorption rates were in accordance with the resorption activities found for TMP and TCP (Fig. 9). The Ca^2+^ level during osteoclast culture for TMP-D and TMP-P increased compared to the cell-free degradation study (Fig. 10b and c), which indicated a cell-mediated partial (TMP-D) or even complete (TMP-P) dissolution of the CHA layer for TMP-D or TMP-P, respectively (Fig. 10j). A striking feature of all scaffold variants was the early onset of cellular resorption beginning on day 0 of osteoclast differentiation and increasing on day 3, which proved a participation of preosteoclasts in bioresorption *in vitro*. This was probably due to the release of metabolic products, which locally reduced the pH and thus resulted in the chemical dissolution of amorphous CHA.

The development of Mg^2+^ and Ca^2+^ concentrations during the culture of TCP scaffolds showed almost identical adsorption and bioresorption kinetics (Fig. 10a and d), which indicated the precipitation and resorption of an Mg^2+^-doped HA layer. In the case of the TMP scaffolds, the Mg^2+^ concentration was significantly higher than the initial Mg^2+^-level of the culture medium and the physiological Mg^2+^ concentration in serum ((0.75 mmol/L - 0.95 mmol/L) [64] (Fig. 10e and f). This illustrated the high chemical solubility of struvite and newberyite (labelled in green) under physiological conditions. Due to this, the cells cultured on TMP were exposed to a permanently elevated Mg^2+^-concentration between 2.1 and 6.8 mmol/L. There is agreement that preosteoclasts and osteoclasts tolerate elevated Mg^2+^ concentrations. In particular, Mg^2+^ concentrations below 10 mmol/L even increase the adhesion and the activity of osteoclasts [65], which could be verified in our study. Interestingly, there was no significant difference between the total bioresorption and passive chemical degradation of TMP at any time point, suggesting that there was no active magnesium phosphate resorption. On the contrary, lower Mg^2+^-concentrations were measured in the cell-seeded samples than in the acellular samples for TMP-P, suggesting that the cells inhibited passive degradation by covering the scaffold surface.

The PO_4_^−3^-level in supernatants of cell-free TCP was almost identical to the PO_4_^−3^-level in cell culture media, which resulted from the low chemical solubility of TCP under physiological conditions. However, a cell-mediated resorption of 0.6 mmol/L – 1.0 mmol/L PO_4_^3−^ corresponding to a release rate of 12 μg PO_4_^3−^/scaffold/day could be observed (Fig. 10g). Accordingly, the resorbed material of TCP from day 3 had a (Ca + Mg)/P-ratio of 1.32–1.41. These - compared to HA (Ca/P = 1.67) - lower ratios indicated a further substitution of apatite with carbonate (Ca/(P + C) = 1.3–1.4) [66], which was also coherent with the observed desert-rose-like morphology (Fig. 3a) characteristic for CHA [67]. According to the chemical composition, Mg^2+^ and PO_4_^3−^ ions were released in a 1:1 ratio for TMP-D (MgNH_4_PO_4_·6 H_2_O) and TMP-P (MgHPO_4_·3 H_2_O). Consequently, an NH_4_^+^-concentration in the range of 3–7 mmol/L could be estimated for TMP-D. It can be assumed that the confrontation with ammonium ions was most probably the cause of the inhibited osteoclastogenesis in the case of TMP-D. To the best of our knowledge, the influence of ammonium ions on osteoclast differentiation was not addressed up to now, and future studies should be carried out to basically evaluate the effect.

After 22 days the TMP-P and TMP-D scaffolds lost only 1.9 ± 0.2 wt% and 4.1 ± 0.7 wt% of their initial weights, respectively. Instead, precipitates on TCP increased the weights of the scaffolds by 0.9 ± 0.4 wt% in the same time. Assuming a linear degradation, a complete chemical degradation after 1.5 years would be expected for an implant based on TMP-D, while TMP-P will be dissolved within 3.1 years. However, *in vivo* degradation is a very complex process, which depends on several factors like implant size, porosity, chemical degradability, cellular resorbability as well as implantation side, and, moreover, can only be approximately simulated *in vitro* due to the lack of cell diversity and interaction or tissue ingrowth.

## 4 Conclusions

Magnesium phosphate ceramics are a promising alternative to calcium phosphate based bone substitutes due to their excellent biocompatibility, mechanical stability, and solubility. We investigated the bioactivity, *in vitro* degradation, and bone cell compatibility of 3D powder printed struvite or newberyite based MP, namely TMP-D and TMP-P. In our study, we were able to confirm the cytocompatibility of these materials. The cell activity of human osteoblasts was increased on both MP based materials, compared to the cells cultured on TCP. TMP-D also performed better than the established material TCP in terms of cell viability. However, cell viability was reduced on TMP-P, indicating a mild to moderate cytotoxic response. Furthermore, by detecting an amorphous CHA precipitate on the surface of both MPs, we were able to demonstrate their bioactivity, which was even 17–33% higher compared to the TCP reference. The low crystalline nature of this HA deposit increased cellular resorbability of HA, making it intrinsically a quickly available calcium reservoir *in vivo* and hence a potential initiator of new bone formation. During the direct cultivation of human osteoclasts on MP scaffolds an accelerated osteoclastogenesis was observed for TMP-P compared to TCP, while preosteoclast cell fusion was reduced in the case of TMP-D. It is worth emphasizing that, in contrast to the CHA precipitate, the MP ceramics showed no signs of cellular resorption *in vitro*. Interestingly, osteoclast activities, e.g. TRAP and CA II activity, on MP scaffolds were in the same range or slightly increased compared to osteoclasts on TCP, even though MP was not resorbed by osteoclasts. However, a chemical dissolution of the material could be demonstrated, on which we predict that a complete degradation of a MP based bone implants *in vivo* will occur within 1.5–3.1 years. Considering the increased metabolic activity of osteoblasts, the unaffected or reduced osteoclast formation, and the complete degradation that occurs due to chemical solubility, MP-based ceramics seem to be promising materials for regenerative bone implants and might even lead to rapid bone regeneration. Further studies under *in vivo* conditions will have to be conducted in the future to confirm this hypothesis. Beside this, measured activities of human osteoclasts directly cultured on the scaffolds reinforced the statement, that CA II activity reflects the resorption activity of osteoclasts on a ceramic material. Since this has so far only been confirmed for calcium phosphate based materials, a generalization to ceramic materials and to cells, which are actively resorbing materials, seems now conclusive. Together with the results of the ion release study we can also conclude, that mono- or binuclear osteoclasts are capable of mineral resorption.

## CRediT authorship contribution statement

**Gefel Eugen:** performed the cell experiments and chemical degradation experiments, Formal analysis, figures. **Moseke Claus:** Writing – original draft, manuscript Writing, Writing – review & editing. **Schmitt Anna-Maria:** performed experiments for porosity quantification and cytotoxicity, formal analysis. **Dümmler Niklas:** performed bioactivity experiments and XRD measurements, formal analysis. **Stahlhut Philipp:** FESEM and EDX analysis, Formal analysis. **Ewald Andrea:** contributed to the intellectual input and manuscript review, Writing – review & editing. **Meyer-Lindenberg Andrea:** project funding, Funding acquisition, manuscript review, Writing – review & editing. **Vorndran Elke:** project funding, Funding acquisition, Supervision, concept cell studies, Conceptualization, Writing – original draft, manuscript Writing, Writing – review & editing.

## Declaration of competing interest

The authors declare no conflict of interest. The funders had no role in the design of the study; in the collection, analyses, or interpretation of data; in the writing of the manuscript, or in the decision to publish the results.

## Figures and Tables

**figs1:**
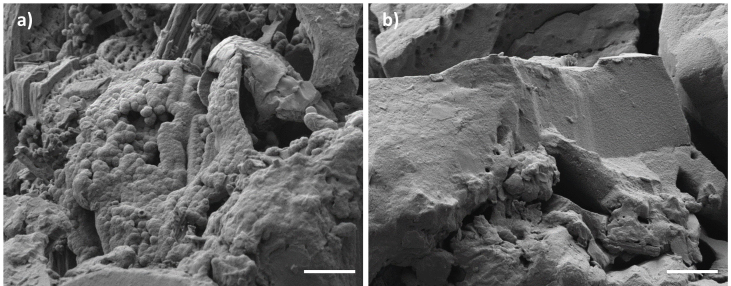


**figs2:**
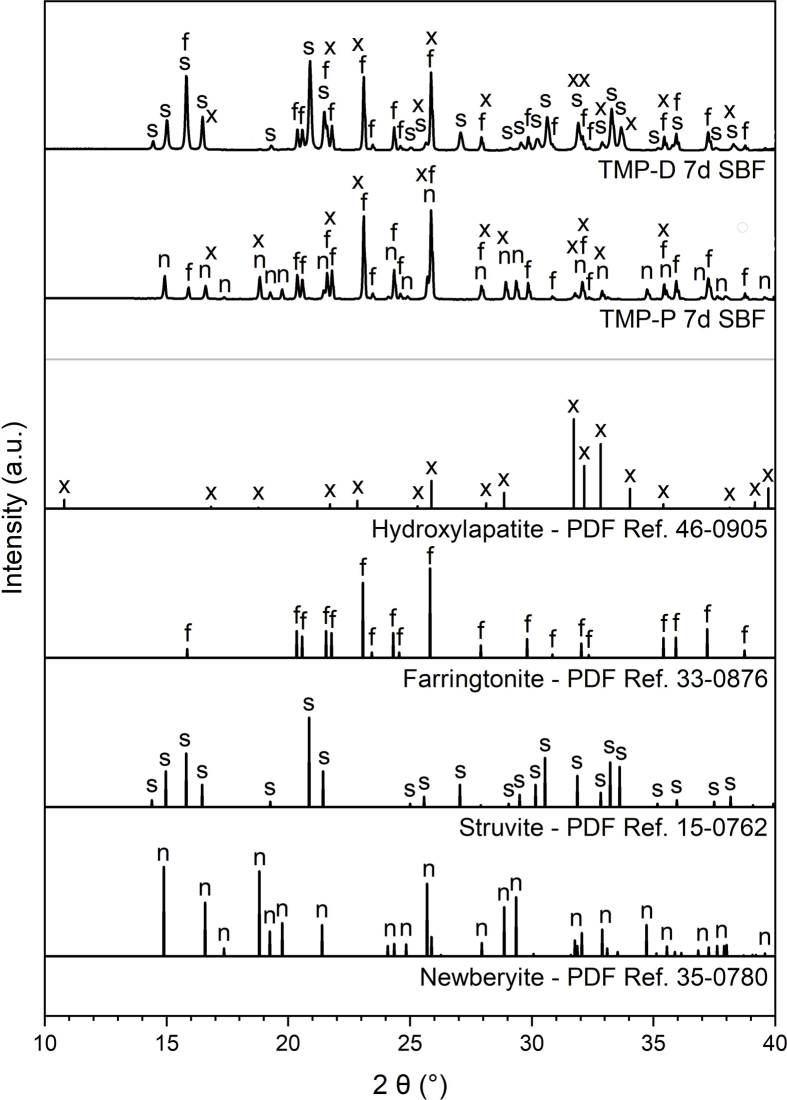


**figs3:**
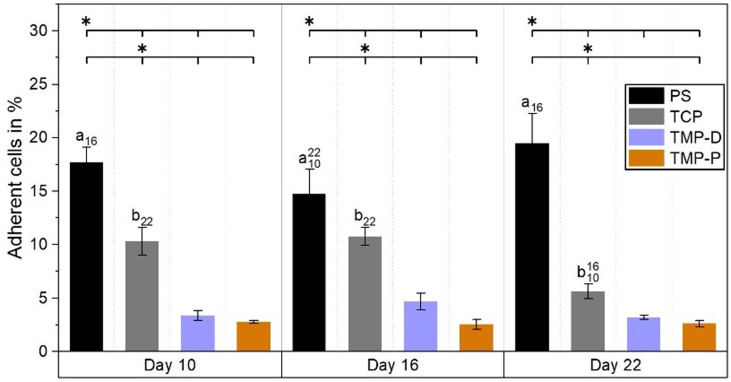


**figs4:**