# Supplementary material for: Degradation of 3D-printed magnesium phosphate ceramics in vitro and a prognosis on their bone regeneration potential
Source: Bioact Mater. 2022 Apr 26;19:376–91. doi: 10.1016/j.bioactmat.2022.04.015 (PMC9062425; doi:10.1016/j.bioactmat.2022.04.015)
Supplement: Multimedia component 1 [file mmc1.docx]

**Supplementary data**


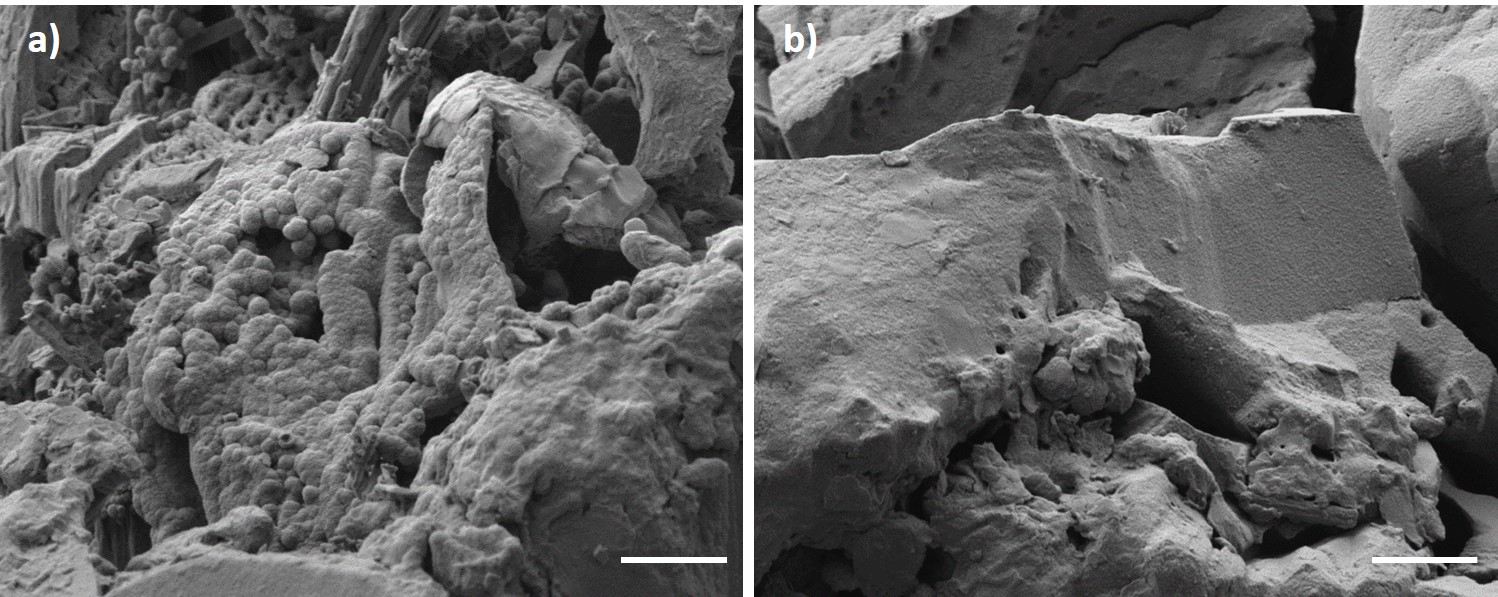


**Figure S1:** a) Spherical precipitates of CHA was detected on TMP-D after immersion for 7 days in SBF. b) The morphology detected by FESEM imaging of TMP-P after 7 days in SBF indicated no precipitation of CHA. Consequentely, the use of FESEM alone was not sufficiently informative for the determination of a very low amount of precipitated CHA. Scale bar = 2 µm.


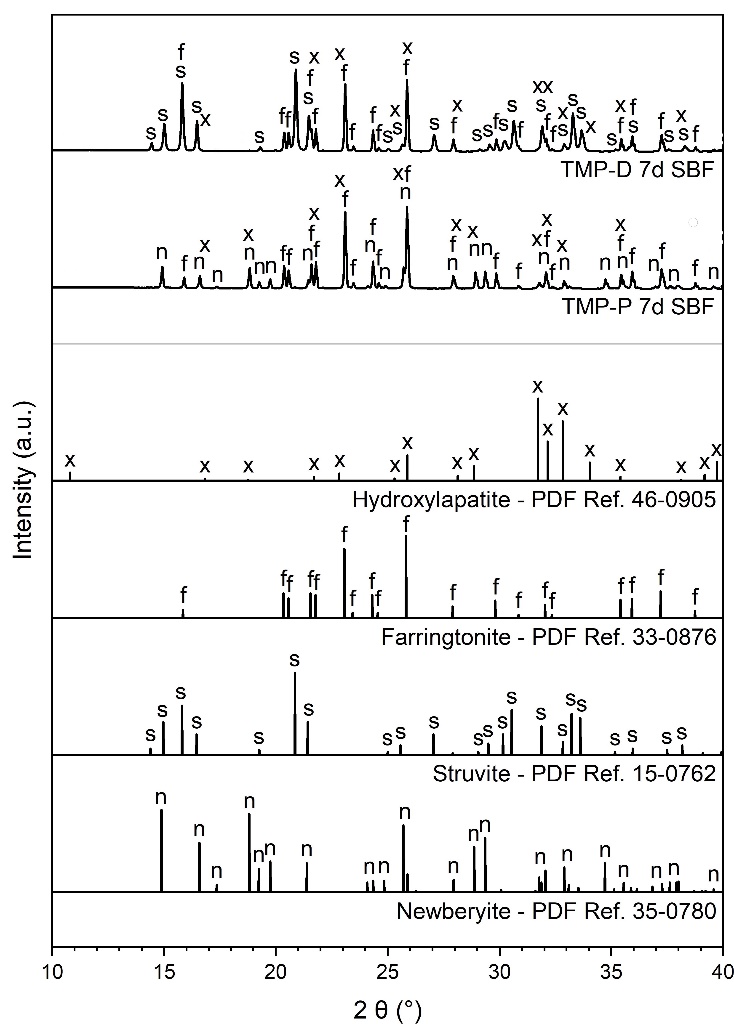


**Figure S2:** Chemical composition of TMP-P and TMP-D after immersion in SBF for 7 days. Hydroxyapatite precipitates cannot be clearly identified by XRD because of overlapping reference patterns and low crystallinity of HA. Newberyite (n), farringtonite (f), struvite (s), hydroxyapatite (x).


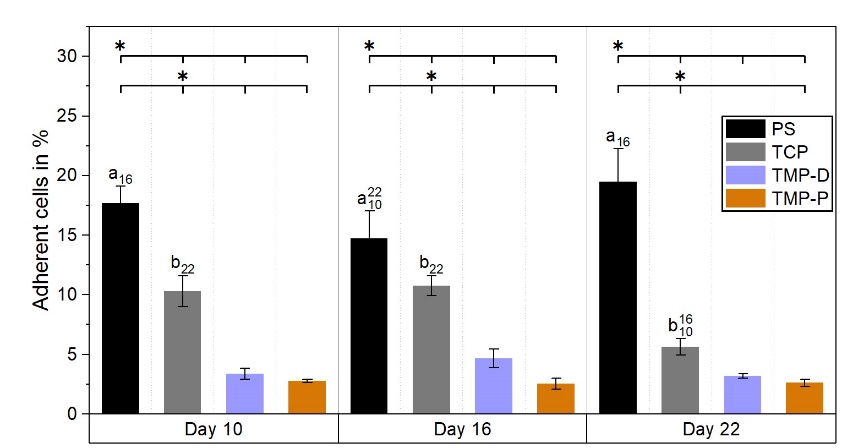


**Figure S3:** Human monocyte cell adhesion on the cell culture control PS and on the scaffolds TCP, TMP-D, and TMP-P. The adherent cells were calculated on the base of DNA content of initially seeded cells and DNA content determined for PS or the scaffolds (without cells grew on the well plate bottom) at day 10, 16, and 22.


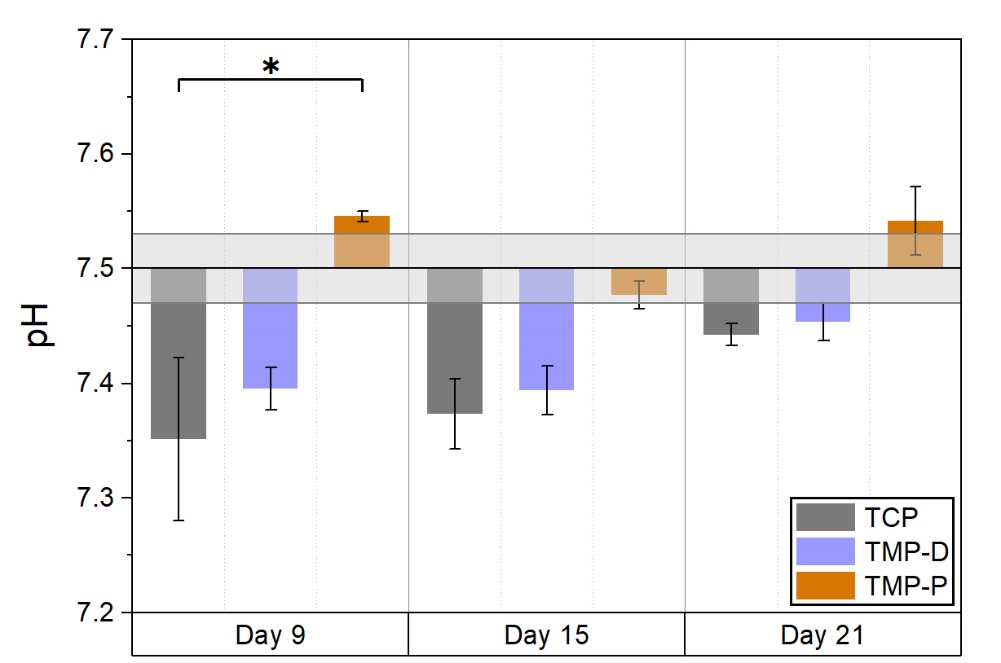


**Figure S4:** pH values during culture of osteoclasts on TCP, TMP-D, and TMP-P scaffolds. There was no significant difference between the pH of pure culture medium (gray shaded) and pH during culture of osteoclasts on scaffolds.
